# Evaluation of the Systemic Inflammatory Landscape and C1q/TNF-Related Protein Profiles in Facial Paralysis

**DOI:** 10.3390/cimb48050517

**Published:** 2026-05-15

**Authors:** Ergin Bilgin, Deniz Baklacı, Tuba Çandar

**Affiliations:** 1 Department of Otolaryngology, Faculty of Medicine, Zonguldak Bulent Ecevit University, 67100 Zonguldak, Turkey; deniz.baklaci@beun.edu.tr; 2 Department of Medical Biochemistry, School of Medicine, Ufuk University, 06520 Ankara, Turkey; tuba.candar@ufuk.edu.tr

**Keywords:** facial paralysis, CTRP3, neutrophil-to-lymphocyte ratio (NLR), adipokines, systemic inflammation

## Abstract

*Background and Objectives*: The primary objective of this study was to elucidate the diagnostic significance of systemic inflammatory indices and C1q/TNF-related proteins (CTRP3 and CTRP5) in acute facial paralysis, with a focus on identifying independent metabolic and immunological predictors. *Materials and Methods*: This retrospective analysis included 64 individuals (25 patients with acute facial paralysis and 39 healthy controls). Complete blood count–derived indices (neutrophil-to-lymphocyte ratio [NLR] and platelet-to-lymphocyte ratio [PLR]), serum albumin, and serum CTRP3/CTRP5 levels (ELISA) were compared between groups. Independent predictors were assessed using binary logistic regression, and discriminative performance was evaluated by ROC analysis. *Results:* Compared with controls, the facial paralysis group showed lower albumin (*p* < 0.001) and lymphocyte counts (*p* = 0.010), higher neutrophil counts (*p* = 0.012), and elevated NLR (*p* < 0.001) and PLR (*p* = 0.026). CTRP3 levels were significantly lower in patients (*p* = 0.002), whereas CTRP5 did not differ between groups (*p* = 0.853). In a parsimonious multivariable logistic regression model adjusted for NLR and CTRP3, NLR remained the only independent predictor (OR = 2.54, *p* = 0.004). ROC analysis showed an AUC of 0.753 for NLR and a raw AUC of 0.265 for CTRP3 (*p* = 0.002). An AUC significantly below 0.5 indicates that lower CTRP3 levels are associated with facial paralysis, which is statistically equivalent to an inverse predictor. *Conclusions*: Acute facial paralysis is characterized by a systemic inflammatory shift and reduced CTRP3 levels. Given the modest sample size, these findings should be strictly considered hypothesis-generating, suggesting that the depletion of anti-inflammatory defenses and increased inflammatory burden may play a role in the acute phase.

## 1. Introduction

Facial paralysis is defined as a complex clinical condition resulting from the loss of voluntary muscle movement due to facial nerve injury, which carries significant aesthetic, functional, and psychological burdens [1,2]. The etiology of this pathology is diverse, ranging from developmental origins and idiopathic cases, most commonly Bell’s palsy, to trauma and neoplastic infiltration [3,4,5]. While the nomenclature and diagnostic criteria have been standardized through international expert consensus, the underlying molecular and systemic inflammatory mechanisms that govern the severity and recovery of the condition remain a subject of intense investigation [2,6,7]. Given its profound psychosocial impact on patient quality of life, the identification of reliable prognostic and diagnostic indicators is considered a clinical priority [5,8].

The systemic inflammatory response is hypothesized to play a critical role in the pathophysiology of peripheral nerve injuries [9,10]. In recent years, hematological indices derived from routine blood counts, such as the Neutrophil-to-Lymphocyte Ratio (NLR) and Platelet-to-Lymphocyte Ratio (PLR), have been recognized as cost-effective and accessible markers of systemic stress and immune balance [10,11]. NLR serves as a proxy for the equilibrium between innate immunity and adaptive immunity, while PLR reflects interactions between thrombotic pathways and inflammatory cytokine loads [11,12]. These markers have demonstrated significant diagnostic value in distinguishing between central and peripheral facial paralysis and are increasingly evaluated as prognostic factors in both adult and pediatric populations [12,13,14].

Beyond cellular immune markers, the role of adipocyte-derived signaling molecules in neural homeostasis has gained prominence [15,16]. The C1q/TNF-related protein (CTRP) family, a group of structurally complex proteins related to adiponectin and the C1q complement component, is identified as a significant regulator of metabolic and inflammatory pathways [16,17]. Among these, CTRP3 is highlighted for its multifunctional roles, particularly its anti-inflammatory properties and its capacity to regulate glucose metabolism [18,19]. Conversely, CTRP5 has been associated with energy balance through AMPK activation, although its regulation in the context of adipocyte biology is specifically linked to metabolic and cellular stress [20,21]. The specific interplay between these CTRP hormones and the systemic inflammatory profile in the context of acute nerve damage has not been fully elucidated in existing literature [15,17].

Despite the established use of NLR and PLR as inflammatory markers, their integration with novel metabolic biomarkers such as CTRP3 and CTRP5 in facial paralysis research remains limited. A comprehensive understanding of the systemic signature of this condition is necessary to refine diagnostic accuracy and develop targeted therapeutic strategies. This exploratory study was conducted to evaluate the potential diagnostic significance of CTRP3, CTRP5, and hematological indices (NLR and PLR) in patients with acute facial paralysis. The scope of this research encompasses comparing these biomarkers between patient and control groups to determine their potential as independent markers associated with the condition. It is anticipated that these findings will contribute to the preliminary molecular characterization of facial paralysis and provide a hypothesis-generating foundation for the clinical stratification of affected individuals.

## 2. Materials and Methods

### 2.1. Study Design and Ethical Approval

This study was designed as a retrospective case–control study and was conducted in accordance with the Declaration of Helsinki. Ethical approval was obtained from the Zonguldak Bulent Ecevit University Non-Invasive Clinical Research Ethics Committee (Approval Date: 2021; Decision No: 13). All data were processed in compliance with confidentiality and ethical research standards. Study Population The study cohort consisted of a total of 64 participants. The study cohort consisted of 64 participants enrolled consecutively at a single tertiary academic center. The patient group included 25 individuals diagnosed with acute facial paralysis who presented to our otolaryngology clinic. To ensure a robust comparative baseline, 39 healthy controls were selected from volunteers with no known history of neurological, autoimmune, or systemic inflammatory diseases who were evaluated during the same study period. Participants in the control group were age- and sex-matched to the clinical group to minimize selection bias and ensure baseline comparability.

Strict exclusion criteria were applied to both groups to ensure data integrity. Individuals with a history of recurrent facial paralysis, recent head or neck trauma, pregnancy, or known malignancy were excluded. To minimize confounding effects on CTRP3 and CTRP5 levels, patients with uncontrolled diabetes, morbid obesity (BMI > 35 kg/m^2^), or active autoimmune disorders were also excluded. Additionally, patients with chronic systemic inflammatory diseases or those currently under medication that could interfere with hematological indices were excluded to ensure that the observed fluctuations were primarily associated with the acute phase of facial paralysis. Furthermore, all blood samples were obtained within the first 72 h of symptom onset and prior to the administration of systemic corticosteroids or antiviral agents.

### 2.2. Data Collection and Biochemical Parameters

Demographic data, including age and gender, were recorded for all participants. Venous blood samples were collected to evaluate baseline hematological and biochemical profiles. The primary laboratory parameters analyzed included:

• Complete Blood Count (CBC) indices: Hemoglobin (g/dL), absolute lymphocyte count (10^3^/µL), absolute neutrophil count (10^3^/µL), and platelet count (cells/µL; converted to 10^3^/µL for PLR calculation).
• Biochemical markers: Serum albumin values were initially recorded in g/L and reported in g/dL to ensure consistency with standard clinical reference ranges. Similarly, platelet counts were expressed in 10^3^/µL for PLR calculation and tabulation. Crucially, all primary statistical analyses were conducted using the raw laboratory values to eliminate any potential for transcription or rounding errors during unit conversion.

### 2.3. Metabolic and Genetic Markers: CTRP3 and CTRP5

Serum levels of C1q/tumor necrosis factor-related protein 3 (CTRP3) and C1q/tumor necrosis factor-related protein 5 (CTRP5) were quantified using the Enzyme-Linked Immunosorbent Assay (ELISA) method. These proteins, members of the C1q/TNF-related protein family, share structural homology with adiponectin and serve as critical modulators of metabolic and inflammatory pathways. While CTRP3 is primarily recognized for its anti-inflammatory properties and its role in suppressing hepatic glucose production, CTRP5 is involved in energy homeostasis via AMPK activation and has been linked to both retinal health and metabolic risk profiles.

The measurements were performed using Human C1QTNF3 (Catalog No: E3127Hu) and C1QTNF5 (Catalog No: E4078Hu) ELISA kits from Bioassay Technology Laboratory (BT Lab, Shanghai, China) on a HEALES MB-580 microplate reader. All results were reported in nanograms per milliliter (ng/mL). The precision of the assays was verified by coefficient of variation (CV) values:

• For CTRP3, the intra-assay and inter-assay CV values were 6.43% * and 4.44%, respectively.
• For CTRP5, the intra-assay and inter-assay CV values were 7.24% * and 4.33%, respectively.

* While the intra-assay CV was slightly above the conventional 5% threshold, it was deemed acceptable within the analytical limits provided by the manufacturer for these specific adipokines.

The analytical sensitivity of the ELISA kits was carefully considered. For CTRP3, the minimum detectable dose is 0.05 ng/mL, ensuring that all clinical samples, including those in the lower range, were measured with high precision within the assay’s linear range. The assays were conducted in strict adherence to the manufacturer’s standardized protocols for human serum samples to minimize potential matrix interference.

The proteins investigated in this study are structurally related to C1q, the initiating component of the classical complement pathway. The proteins investigated in this study, CTRP3 and CTRP5, are members of the C1q/TNF-related protein family and share significant structural homology with the C1q molecule. By analyzing these signaling molecules, this study explores how these adipokines reflect the metabolic and inflammatory landscape in patients with facial paralysis. By analyzing CTRP3 and CTRP5, this study aims to explore how these C1q-related signaling molecules reflect the metabolic and inflammatory landscape in patients with facial paralysis.

### 2.4. Inflammatory Indices: NLR and PLR

To assess the systemic inflammatory burden, two pivotal composite indices were calculated from the raw hematological data:

• Neutrophil-to-Lymphocyte Ratio (NLR): Calculated by dividing the absolute neutrophil count by the absolute lymphocyte count.
• Platelet-to-Lymphocyte Ratio (PLR): Calculated by dividing the absolute platelet count by the absolute lymphocyte count.

These ratios were utilized as objective markers to reflect the balance between innate and adaptive immune responses. In the context of facial paralysis, NLR and PLR serve as indirect indicators of systemic physiological stress and the intensity of the inflammatory cascade associated with neural injury.

### 2.5. Statistical Analysis

Statistical analyses were performed using IBM SPSS Statistics 25.0. Before conducting inter-group comparisons, the distributional characteristics of all continuous variables were rigorously assessed using the Shapiro–Wilk test. This fundamental step determined the selection of either parametric or non-parametric analytical tools.

For variables following a normal distribution (*p* > 0.05), the Independent Samples t-test was employed to compare the means of the two groups, and the homogeneity of variances was confirmed via Levene’s test. Conversely, the Mann–Whitney U test was used for variables that did not meet the normality assumption (*p* < 0.05), such as age, NLR, and CTRP levels, to ensure a robust evaluation of median differences. Categorical data, including gender distribution, were analyzed using the Chi-square test to assess baseline comparability between the cohorts.

The relationship between biochemical markers and clinical parameters was explored using Spearman’s rank correlation coefficient, chosen for its suitability with non-normally distributed data. To identify the independent predictors of facial paralysis while avoiding overfitting due to the modest sample size (*n* = 25 cases), a parsimonious Binary Logistic Regression model was constructed. To maintain a robust Events Per Variable (EPV) ratio and ensure stable Odds Ratio (OR) estimates, the model included only those variables that demonstrated strong significance in univariate analysis (NLR and CTRP3). This resulted in an EPV ratio of 12.5 (25 events for 2 predictors), which aligns with the recommended methodological threshold of 10–15 events per variable to prevent model instability. The performance and significance of the model were evaluated using the Omnibus test, and the coefficient of determination was reported through Cox & Snell R2 and Nagelkerke R2 values. The calibration of the logistic regression model was assessed using the Hosmer-Lemeshow goodness-of-fit test, where a *p*-value > 0.05 was interpreted as an indicator of good model calibration, confirming that the model’s predicted probabilities were well-aligned with the observed clinical outcomes.

Finally, the diagnostic accuracy, sensitivity, and specificity of the investigated biomarkers were determined using Receiver Operating Characteristic (ROC) curve analysis. The Area Under the Curve (AUC) values were calculated to quantify the discriminative power of each marker. All statistical tests were two-tailed. To minimize the risk of Type I errors arising from multiple univariate comparisons of laboratory parameters, a Bonferroni correction was applied. For these 11 specific comparisons, a *p*-value of less than 0.0045 (0.05/11) was established as the threshold for statistical significance. For all other analyses, a *p*-value of less than 0.05 was maintained as the standard level of significance. Due to the retrospective nature of the study, an a priori power analysis was not conducted. Consequently, this study is presented as an exploratory, hypothesis-generating investigation. To evaluate the adequacy of the sample size, post hoc effect sizes (r) were calculated for the primary outcome measures, where values of 0.1, 0.3, and 0.5 were considered small, medium, and large effects, respectively.

## 3. Results

### 3.1. Demographic and Clinical Characteristics

The demographic and baseline clinical parameters of the study participants are presented in Table 1. The final analysis included 64 subjects, with 39 individuals in the healthy control group and 25 patients in the facial paralysis group. Initial evaluations focused on the homogeneity of the cohorts to ensure balanced comparisons for the primary biomarkers. Due to the retrospective nature of the clinical records, baseline comorbidities (diabetes, hypertension) and smoking status were not systematically documented and are acknowledged as missing variables.

### 3.2. Statistical Analysis

Initially, the distributional properties of all continuous variables were rigorously assessed using the Shapiro–Wilk normality test to determine the appropriate statistical framework. According to the test results, the age variable did not follow a normal distribution in either the control or facial paralysis groups (*p* < 0.05). Conversely, hemoglobin levels were found to conform to a normal distribution across both cohorts (*p* > 0.05). Other key laboratory parameters, including albumin, lymphocyte, platelet, and neutrophil counts, along with CTRP3 and CTRP5 levels, failed to meet the normality assumption in at least one of the groups (*p* < 0.05). Consequently, the selection of parametric or non-parametric tests for inter-group comparisons was strictly based on these individual distribution profiles.

The demographic comparability of the groups was verified through specific analyses. The Chi-square test revealed no statistically significant difference in gender distribution between the cohorts (*p* > 0.05), indicating that the groups were comparable in terms of biological sex. Regarding age, although the facial paralysis group exhibited a higher median age compared to the control group, this difference was further evaluated using the Mann–Whitney U test for statistical significance.

Preliminary biochemical observations indicated distinct trends between the groups. The healthy control group was characterized by higher albumin and lymphocyte concentrations, whereas a lower neutrophil count was recorded. In contrast, the facial paralysis group showed an upward trend in neutrophil levels, reflecting a potential acute inflammatory response. Notably, while CTRP3 concentrations were markedly higher in the control subjects, they were found to be substantially lower in patients with facial paralysis. Conversely, CTRP5 levels showed an inclination to increase in the paralysis cohort. To evaluate these differences, the Independent Samples t-test was utilized for normally distributed variables, and the Mann–Whitney U test was applied to those with skewed distributions. Furthermore, correlation analyses were performed using either Pearson or Spearman coefficients, dictated by the normality of the relevant data sets.

### 3.3. Comparative Analysis of Laboratory and Inflammatory Markers

The comparative analysis of hematological and biochemical markers between the study groups revealed significant physiological shifts (Table 2). To ensure statistical rigor, the Shapiro–Wilk test was applied to the NLR and PLR. The results indicated a non-normal distribution for NLR in both the control (*p* = 0.002) and patient groups (*p* < 0.001). Similarly, PLR violated the normality assumption in the control group (*p* < 0.001), although it remained borderline in the facial paralysis cohort (*p* = 0.055). Given these skewed distributions and the presence of outliers, non-parametric methodologies were preferred for subsequent group comparisons.

The study included 39 controls and 25 patients with facial paralysis (Table 1). Age did not differ significantly between groups (36.0 [26.0–48.5] vs. 43.0 [27.0–66.0] years; *p* = 0.112), and sex distribution was comparable (female 61.5% vs. 44.0%; *p* = 0.204). Hemoglobin levels were similar between groups (13.62 ± 1.76 vs. 13.26 ± 1.39 g/dL; *p* = 0.368).

Following the application of the Bonferroni correction (adjusted alpha = 0.0045) to account for multiple comparisons, the facial paralysis group exhibited significantly lower albumin (4.50 [4.30–4.70] vs. 4.80 [4.55–5.00] g/dL; *p* < 0.001) and NLR levels were significantly higher in patients (2.93 [2.12–5.09] vs. 1.91 [1.36–2.59]; *p* < 0.001). While lymphocyte counts (*p* = 0.010), neutrophil counts (*p* = 0.011), and PLR (*p* = 0.026) showed numerical differences, these did not reach the adjusted threshold for statistical significance. Platelet counts showed no difference (*p* = 0.390). Regarding the C1q/TNF-related protein family, CTRP3 was significantly lower in the facial paralysis group (0.50 [0.23–2.51] vs. 2.84 [1.54–3.08] ng/mL; *p* = 0.002), maintaining its significance after Bonferroni adjustment. Conversely, CTRP5 levels showed no statistically significant difference between the cohorts (*p* = 0.853).

These findings highlight CTRP3 and NLR as the primary biochemical indicators in this cohort. The marked reduction in CTRP3, alongside decreased albumin, may reflect the protein’s known anti-inflammatory and metabolic regulatory roles, constituting a distinct biochemical profile for acute facial paralysis.

### 3.4. Correlation Analysis of Biochemical and Clinical Parameters

The interrelationships between the evaluated variables were scrutinized using Spearman’s rank correlation analysis, as detailed in Table 3. This analysis aimed to uncover potential associations between metabolic markers, inflammatory indices, and demographic data.

A significant, moderate negative correlation was identified between Albumin and Age (ρ = −0.434, *p* < 0.001), suggesting that albumin levels tend to decline with advancing age within the study population. Notably, a statistically significant positive correlation, albeit of weak-to-moderate strength, was observed between Albumin and CTRP3 levels (ρ = 0.269, *p* = 0.032). This finding indicates that CTRP3 concentrations may fluctuate in parallel with nutritional status and anti-inflammatory capacity.

Furthermore, a robust and predictable strong positive relationship was detected between Neutrophils and the NLR (ρ = 0.748, *p* < 0.001), reinforcing the NLR’s utility as a direct reflection of neutrophil-driven systemic inflammation. Interestingly, CTRP5 did not exhibit any statistically significant correlation with the investigated parameters (*p* > 0.05). These results suggest that while CTRP3 is integrated into the nutritional-inflammatory axis, CTRP5 may follow a more independent or heterogeneous distribution pattern within this specific clinical sample.

### 3.5. Binary Logistic Regression Analysis

A Binary Logistic Regression model was constructed to evaluate the potential of various parameters in predicting the occurrence of facial paralysis (Control = 0, Facial Paralysis = 1). The final multivariable model was restricted to NLR and CTRP3 to ensure statistical stability and minimize the risk of overfitting given the cohort size. To maintain a robust Events Per Variable (EPV) ratio, the inclusion of two predictors for 25 cases resulted in an EPV of 12.5, which aligns with the recommended methodological threshold of 10–15 to prevent model instability. The overall model demonstrated statistical significance according to the Omnibus test ($X^{2}$ = 25.197, *p* < 0.001). The explained variance was represented by a Cox & Snell $R^{2}$ of 0.325 and a Nagelkerke $R^{2}$ of 0.441. The model’s overall goodness-of-fit was validated using the Hosmer-Lemeshow test ($X^{2}$ = 6.046, *p* = 0.642), where a *p*-value > 0.05 indicates good model calibration, confirming that the model’s predicted probabilities are well-aligned with the observed clinical outcomes.

Upon examining the specific contributions of each variable, as summarized in Table 4, the NLR emerged as the only significant independent predictor of facial paralysis (*B* = 0.933, *p* = 0.004). The odds ratio for NLR was Exp(B) = 2.543 (95% CI: 1.347–4.802), indicating that each one-unit increase in NLR was associated with a 2.54-fold higher odds of facial paralysis (*p* = 0.004). Conversely, demographic factors such as age and gender did not reach statistical significance within this multivariate context (*p* > 0.05).

While CTRP3 showed a significant difference in initial univariate comparisons, its independent predictive power was attenuated in the multivariate model (*p* = 0.152). This shift suggests that the influence of CTRP3 may be mediated by broader inflammatory patterns, positioning it as a concomitant biomarker associated with the inflammatory and metabolic landscape rather than a standalone diagnostic tool. This highlight underscores the dominant role of NLR as a primary indicator of systemic stress in facial paralysis patients.

### 3.6. Diagnostic Performance and ROC Curve Analysis

The diagnostic performance of the investigated biomarkers was evaluated using Receiver Operating Characteristic (ROC) curve analysis. The performance metrics, including the Area Under the Curve (AUC), 95% confidence intervals, and statistical significance for each parameter, are detailed in Table 5.

The NLR demonstrated the most robust diagnostic performance among the variables, with an AUC of 0.753 (95% CI: 0.625–0.882; *p* = 0.001). Using the Youden index, the optimal cut-off value for NLR was determined to be 2.81, yielding a sensitivity of 56.0% and a specificity of 89.7%. This indicates that NLR possesses significant discriminative power for identifying acute facial paralysis. Post hoc analysis of effect sizes (r) further confirmed that the study was sufficiently powered to detect meaningful differences in primary markers, specifically for NLR (r = 0.48), Albumin (r = 0.43), and CTRP3 (r = 0.39).

**Figure 1:**
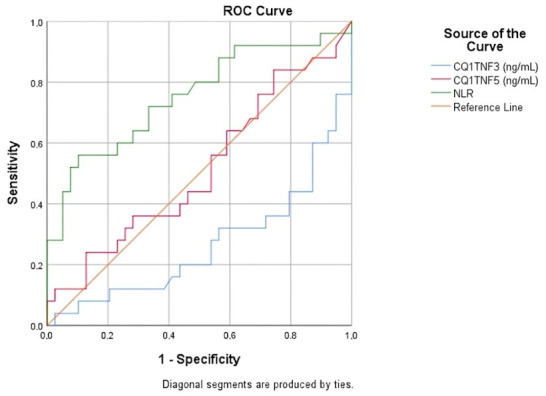
Receiver Operating Characteristic (ROC) curves of NLR, CTRP3, and CTRP5 for the prediction of facial paralysis. NLR shows the highest diagnostic accuracy, while CTRP3 demonstrates a significant inverse association. Note: Receiver Operating Characteristic (ROC) curves evaluating the diagnostic performance of NLR and CTRP3. The X-axis represents the 1-specificity (False Positive Rate), and the Y-axis represents sensitivity (True Positive Rate). (A) NLR curve demonstrates the predictive power for facial paralysis. (B) CTRP3 curve reflects the inverse relationship with the disease state.

For CTRP3, the ROC analysis (Figure 1) yielded a raw AUC of 0.265 (95% CI: 0.137–0.393, *p* = 0.002). An AUC significantly below 0.5 indicates that lower CTRP3 levels are characteristically associated with facial paralysis, which is statistically equivalent to an inverse predictor (inverted AUC = 0.735). Using the Youden index based on this relationship, the optimal cut-off for CTRP3 was identified as 1.21 ng/mL, providing a sensitivity of 64.0% and a specificity of 79.5%. Conversely, CTRP5 did not show any standalone diagnostic value, achieving an AUC of 0.514 (*p* = 0.853).

### 3.7. Synthesis of Inflammatory and Metabolic Indicators

The collective data suggests a distinct shift in the systemic inflammatory and metabolic landscape in facial paralysis patients. The concurrent reduction in Albumin and Lymphocytes, coupled with elevated Neutrophils and NLR, highlights a dominant inflammatory response. The integration of NLR and PLR as cost-effective markers reflects the balance between innate and adaptive immunity, which appears to be significantly disrupted in these cases.

The multivariable logistic regression model demonstrated robust calibration, as confirmed by the Hosmer-Lemeshow test (Chi-square = 6.046, *p* = 0.642), indicating no significant lack of fit between the model and the observed clinical data. The emergence of NLR as the sole independent risk factor where each unit increase elevates the probability of facial paralysis by 2.5 times underscores the importance of routine hematological indices in clinical assessment. In summary, while CTRP3 serves as a significant concomitant metabolic marker, NLR stands out as the primary independent indicator of systemic stress and neural injury in this population.

## 4. Discussion

The clinical landscape of acute facial paralysis necessitates the identification of biomarkers that can accurately reflect the underlying pathophysiological processes of nerve injury and systemic stress. This research provides significant evidence regarding the metabolic and inflammatory signatures of the condition, emphasizing the roles of the NLR and CTRP3. The findings suggest that while multiple parameters fluctuate during the acute phase, certain indices possess superior predictive power and independent diagnostic value.

A primary finding of this study is the marked elevation of systemic inflammatory markers in patients with facial paralysis compared to healthy controls. Specifically, the numerical increase in neutrophil counts and the decrease in lymphocyte levels, while reflecting a clear trend, contributed to the substantially higher NLR and PLR values resulted in substantially higher NLR and PLR values in the patient group. These indices are recognized as cost-effective reflectors of the balance between innate and adaptive immune responses [10,11]. In this study, NLR emerged as a significant biological marker associated with facial paralysis. While our model suggests a 2.5-fold increase in risk per unit, this finding should be interpreted as a potential indicator of inflammatory burden rather than a definitive independent predictor, given the inherent statistical variability of our sample size. This observation is consistent with recent meta-analyses and clinical studies that highlight NLR as a robust indicator of the inflammatory burden in Bell’s palsy and other peripheral neuropathies [10,12,13]. The superiority of NLR over isolated leukocyte or platelet counts suggests that the relative shift in immune cell populations provides a more sensitive measure of the systemic stress associated with facial nerve dysfunction.

Furthermore, the investigation of the CTRP family revealed a significant reduction in CTRP3 levels among patients with facial paralysis. CTRP3 is known for its potent anti-inflammatory and metabolic regulatory functions [15,18]. The observed inverse relationship, supported by a raw AUC of 0.265, reinforces the hypothesis that CTRP3 acts as a protective or anti-inflammatory agent that is depleted during the acute phase of the disease. An AUC significantly below 0.5 indicates that lower CTRP3 levels are associated with facial paralysis, which is statistically equivalent to an inverse predictor. We identified a weak-to-moderate positive correlation between CTRP3 and albumin levels (ρ = 0.269, *p* = 0.032). While the low coefficient of determination (R^2^ = 0.072) suggests that this relationship explains only a small fraction of the variance, it may still point toward a shared biochemical axis involving nutritional status and anti-inflammatory pathways [18,19]. However, this preliminary finding requires validation in larger cohorts to ensure it is not a result of multiple testing. Interestingly, although CTRP3 showed strong discriminative power in univariate comparisons, its significance was attenuated in the multivariate logistic regression model. This phenomenon can be attributed to the dominant statistical effect of NLR, suggesting that while CTRP3 is a relevant biochemical marker, its fluctuations are likely concomitant with the broader systemic inflammatory response rather than acting as a standalone diagnostic driver. From a clinical perspective, the simultaneous assessment of CTRP3 and NLR may offer a practical, objective framework for evaluating the inflammatory and metabolic burden in patients with acute facial paralysis. While NLR indicates the severity of the systemic inflammatory response, the depletion of CTRP3 provides a molecular window into the compromised anti-inflammatory defenses during the acute phase. The anti-inflammatory role of CTRP3 in acute neural injury may be further understood through its interaction with intracellular signaling cascades, particularly the Mitogen-Activated Protein Kinase (MAPK) and PI3K/Akt/mTOR pathways. MAPK signaling is a central mediator in immunity and systemic inflammation, serving as a bridge between extracellular stimuli and the pro-inflammatory cytokine response [22]. CTRP3 has been shown to modulate these signaling pathways, potentially reducing pro-inflammatory signals. Simultaneously, the activation of the PI3K/Akt pathway and its downstream effector mTOR plays a crucial role in orchestrating immune defense and cellular survival mechanisms [23]. The depletion of CTRP3 observed in our cohort may represent a compromise in these protective signaling axes, potentially exacerbating the inflammatory-neural damage in facial paralysis.

In contrast to CTRP3, CTRP5 levels did not exhibit statistically significant differences between the cohorts, nor did they show independent predictive value. While CTRP5 is structurally related to other CTRP members and involved in energy homeostasis, its role in acute neural injury appears less distinct in this specific sample [17,20]. This heterogeneity suggests that the adipokine response in facial paralysis is not uniform across all family members, with CTRP3 being a more reliable indicator of the metabolic-inflammatory state in this context.

The observed correlations also provide insight into the metabolic background of the participants. The moderate negative correlation between albumin and age, along with the patterns of inflammatory indices, aligns with the established understanding of how aging and systemic health status influence recovery and susceptibility to neural insults [9,21]. Given the retrospective design and modest sample size (*n* = 25 cases), these findings should be strictly considered hypothesis-generating and require validation in larger prospective cohorts. This scale constrained the number of variables we could include in the multivariable logistic regression without risking overfitting. While the lack of an a priori power calculation is a limitation, the post hoc assessment suggests that our cohort was adequate to identify these specific inflammatory and metabolic shifts. However, we explicitly acknowledge that our results serve as a preliminary foundation rather than definitive clinical proof. Another limitation is the lack of systematic Body Mass Index (BMI) and smoking status data. Given that CTRP3 is an adipokine, the absence of BMI-adjusted analyses is a significant factor; future research must incorporate body composition metrics to isolate the specific impact of facial paralysis on these protein profiles. It is important to note that the cross-sectional design of this study precludes any definitive claims regarding causality. The observed 2.5-fold increase in the probability of facial paralysis associated with NLR should be interpreted as a statistical association reflecting the systemic stress response, rather than a direct causal driver of the disease. Furthermore, NLR is a non-specific marker of systemic inflammation that can be influenced by various infectious, autoimmune, and metabolic conditions. Consequently, while its high sensitivity makes it a valuable tool for assessing the inflammatory burden in facial paralysis, its clinical utility is best realized as a complementary index alongside clinical evaluation rather than a standalone diagnostic test.

Future Research Directions: Beyond these limitations, our findings pave the way for longitudinal studies that could investigate the potential changes in these markers during the recovery phase. Specifically, monitoring the recovery of CTRP3 levels in parallel with clinical improvements, such as House-Brackmann scores, could determine if these proteins serve as dynamic indicators of therapeutic response or neural regeneration. Such research will be crucial in refining personalized monitoring and treatment protocols for facial nerve disorders.

## 5. Conclusions

This study demonstrates that acute facial paralysis is characterized by a significant systemic inflammatory response and a distinct alteration in metabolic biomarkers. The most compelling finding is the identification of the NLR as a significant marker associated with the condition’s inflammatory state. While our results highlight a statistical link where higher NLR levels correlate with the probability of facial paralysis, this should be viewed as a reflection of systemic stress. Its potential clinical utility as a cost-effective adjunct tool remains a preliminary observation that requires confirmation in broader and more diverse populations to establish its precise diagnostic role. Furthermore, the significant reduction in CTRP3 levels in the patient group, accompanied by a significant inverse relationship in ROC analysis (AUC = 0.265, *p* = 0.002), confirms that depleted CTRP3 levels are a key feature of the acute phase. This AUC value reflects a strong inverse association where lower CTRP3 concentrations are characteristically associated with the disease state. Although CTRP3 remained a significant discriminator in univariate analysis, its significance was attenuated in the multivariable model, potentially due to the predominant statistical impact of NLR. This suggests that CTRP3 fluctuations are deeply integrated into the broader inflammatory-metabolic milieu of the disease. Conversely, CTRP5 showed no diagnostic utility (*p* = 0.853), indicating that the adipokine-related response in facial paralysis may be specific to certain family members like CTRP3. While these results are promising, the study is not without limitations, including its retrospective nature and a relatively focused sample size, which may affect the broader generalizability of the findings. Given the retrospective design and modest sample size, these findings should be considered hypothesis-generating. Future prospective research with larger, multi-center cohorts is warranted to validate these biomarkers and explore their potential as diagnostic indicators and markers of disease severity. Ultimately, integrating routine hematological indices with novel adipokines like CTRP3 offers a more comprehensive framework for the clinical stratification and management of facial nerve disorders. Beyond diagnostic stratification, these biomarkers may reflect the systemic state of acute nerve injury; however, their role in monitoring neural recovery requires exploration in future longitudinal clinical trials.

## Figures and Tables

**Table 1:** The Demographic and Baseline Clinical Characteristics of the Study Participants.

Variables	Control Group (*n* = 39)	Facial Paralysis Group (*n* = 25)
Age (Years)	37.95 ± 2.21	46.48 ± 4.44
Gender (Female/Male)	24/15	11/14
Hemoglobin (g/dL)	13.62 ± 0.28	13.26 ± 0.28
Albumin (g/dL)	4.79 ± 0.04	4.4 ± 0.12
Lymphocyte (10^3^/µL)	2.18 ± 0.13	1.68 ± 0.15
Platelet (10^3^/µL)	265.54 ± 10.42	256.56 ± 13.90
Neutrophil (10^3^/µL)	4.01 ± 0.17	5.61± 0.63
C1QTNF3 (ng/mL)	3.86 ± 1.07	1.88 ± 0.75
C1QTNF5 (ng/mL)	3.20 ± 0.42	3.93 ± 0.84

Note: This table presents the demographic and baseline clinical characteristics of the study participants. Data are expressed as Mean ± Standard Deviation (SD) for continuous variables. The values represent the distribution of hematological and biochemical markers within the healthy control (*n* = 39) and facial paralysis (*n* = 25) groups before statistical inferential analysis. Systematic data regarding Body Mass Index (BMI), smoking status, and specific comorbidities such as diabetes and hypertension were unavailable in the retrospective clinical records and are therefore acknowledged as missing data.

**Table 2:** Statistical Comparison of Clinical and Laboratory Parameters Between Groups.

Variable	Test Method	*p*-Value	Statistical Significance
Age	Mann–Whitney U	0.112	Non-Significant
Gender	Chi-Square Test	0.204	Non-Significant
Hemoglobin	Independent *t*-test	0.368	Non-Significant
Albumin	Mann–Whitney U	<0.001	Significant
Lymphocyte	Mann–Whitney U	0.010	Non-Significant *
Neutrophil	Mann–Whitney U	0.011	Non-Significant *
Platelet	Mann–Whitney U	0.390	Non-Significant
CTRP3	Mann–Whitney U	0.002	Significant
CTRP5	Mann–Whitney U	0.853	Non-Significant
NLR	Mann–Whitney U	<0.001	Significant
PLR	Mann–Whitney U	0.026	Non-Significant *

* Note: After Bonferroni correction (adjusted alpha = 0.0045), these variables did not reach statistical significance.

**Table 3:** Spearman Correlation Matrix of Key Laboratory and Demographic Variables.

Variable Relationship	Correlation Coefficient (ρ)	*p*-Value	Interpretation
Albumin vs. Age	−0.434	<0.001	Significant (Moderate Negative)
Albumin vs. CTRP3	0.269	0.032	Significant (Weak-to-Moderate Positive)
Neutrophil vs. NLR	0.748	<0.001	Significant (Strong Positive)

Note: This table presents Spearman’s rank correlation analysis, which was utilized to evaluate the direction and strength of the linear relationships between key demographic, metabolic, and inflammatory variables. The Correlation Coefficient ρ values indicate the nature of the association: The negative correlation between Albumin and Age ρ = −0.434, *p* < 0.001) reflects a moderate decline in serum albumin levels with increasing age. The positive correlation between Albumin and CTRP3 ρ = 0.269, *p* = 0.032) suggests a weak-to-moderate biochemical link between nutritional status and anti-inflammatory protein levels. The strong positive correlation between Neutrophil counts and NLR (ρ = 0.748, *p* < 0.001) validates the NLR as a highly sensitive reflection of neutrophil-driven systemic inflammation.

**Table 4:** Binary Logistic Regression Model for Predicting Facial Paralysis.

Predictor Variable	B	S.E.	Wald	df	*p*-Value	Exp(B) (Odds Ratio)
NLR	0.933	0.324	8.281	1	0.004	2.543
CTRP3	−0.106	0.074	2.053	1	0.152	0.899
Constant	2.312	1.455	2.523	1	0.112	0.099

Note: NLR emerged as the sole independent predictor (*p* = 0.004), with an Odds Ratio of 2.543 (95% CI: 1.347–4.802). The Hosmer-Lemeshow *p*-value of 0.642 indicates good model calibration.

**Table 5:** Diagnostic Performance Metrics (ROC Analysis) for Key Biomarkers.

Variable	AUC	*p*-Value	Optimal Cut-Off	Sensitivity (%)	Specificity (%)	Clinical Interpretation
NLR	0.753	0.001	≥2.81	56.0	89.7	Good Diagnostic Power
CTRP3 *	0.265 *	0.002	≤1.21	64.0	79.5	Good Discrimination
CTRP5	0.514	0.853	-	-	-	No Discriminative Power

* Note: The raw AUC of 0.265 for CTRP3 reflects a significant inverse association with facial paralysis. The cut-off value of 1.21 ng/mL was determined based on this relationship, where values ≤ 1.21 ng/mL are associated with an increased probability of the disease.

## Data Availability

The original contributions presented in this study are included in the article. Further inquiries can be directed to the corresponding author.
